# Transcriptome-Based Identification of the Optimal Reference Genes for Quantitative Real-Time Polymerase Chain Reaction Analyses of Lingonberry Fruits throughout the Growth Cycle

**DOI:** 10.3390/plants12244180

**Published:** 2023-12-16

**Authors:** Wanchen Zhang, Jian Xu, Qiang Wang, Jing Li, Yadong Li, Mei Dong, Haiyue Sun

**Affiliations:** 1 Joint International Research Laboratory of Modern Agricultural Technology, College of Horticulture, Jilin Agricultural University, Changchun 130118, China; wanchenzhang@foxmail.com (W.Z.); xj362442512@163.com (J.X.); blueberryli@163.com (Y.L.); 2 College of Life Science, Jilin Agricultural University, Changchun 130118, China; 3 Research Institute of Pomology of CAAS, Xingcheng 125100, China; wqiang805@126.com (Q.W.); lijing01@caas.cn (J.L.)

**Keywords:** lingonberry, qRT–PCR, transcriptome, internal reference gene, abiotic stress

## Abstract

(1) Background: *Vaccinium vitis-idaea* is a nutritionally and economically valuable natural wild plant species that produces berries useful for treating various diseases. There is growing interest in lingonberry, but there is limited information regarding lingonberry reference genes suitable for gene expression analyses of different tissues under various abiotic stress conditions. The objective of this study was to identify stable reference genes suitable for different lingonberry tissues in response to abiotic stress. (2) Methods: The delta Ct method and the GeNorm v3.5 and NormFinder v20 programs were used to comprehensively analyze gene expression stability. (3) Results: *Actin Unigene23839* was the best reference gene for analyzing different cultivars, whereas *Actin CL5740.Contig2* was the most suitable reference gene for analyzing different tissues and alkali stress. In contrast, *18S rRNA CL5051.Contig1* was the most stable reference gene under drought conditions. (4) Conclusions: These suitable reference genes may be used in future qRT-PCR analyses of different lingonberry tissues and the effects of abiotic stresses. Furthermore, the study data may be useful for functional genomics studies and the molecular breeding of lingonberry. In summary, internal reference genes or internal reference gene combinations should be carefully selected according to the experimental conditions to ensure that the generated gene expression data are accurate.

## 1. Introduction

*Vaccinium* spp. is a genus with approximately 450 species with a worldwide distribution [1]. This genus includes the following three recently domesticated crops: lingonberry, blueberry, and cranberry. Lingonberry (*V. vitis-idaea* L.) belongs to the rhododendron family (Ericaceae). There is increasing interest in the lingonberry fruit, likely because of its high nutritional, therapeutic, and medicinal value [2]. Lingonberry is a small evergreen perennial shrub that can grow to a height of 25 cm, with spherical berries that have a vivid red color. Because raw lingonberry fruits have an undesirable taste, they are mainly used for processing. Lingonberry plants are highly resistant to drought and cold conditions, making them appropriate for cultivation at high altitudes (up to 3000 m above sea level) and near the Arctic Circle. In China, wild lingonberry plants distributed over large areas in the Greater Khingan Mountains have important uses [3].

In molecular biology research, gene expression profiles provide valuable information regarding gene functions, complex biological processes, metabolic pathways, and physiological responses to stress. Various technologies and tools have been developed for analyzing gene expression, including quantitative real-time PCR(qRT-PCR), semi-quantitative PCR, Northern blotting, high-throughput sequencing, and gene chips [4]. Common reference genes used for gene expression analyses in plants often encode actin [5], β-tubulin [6], and 18S ribosomal RNA (18S) [7]. However, there is no universal reference gene across different species, with the most stable reference genes varying among plant species or treatment conditions [8]. Moreover, there has been relatively little functional genomics research on lingonberry. Previous research confirmed that qRT-PCR is a powerful technique for accurately analyzing gene expression levels in different samples or under diverse experimental conditions in a wide range of biological specimens [9].

Recent studies revealed the inconsistencies in the expression of reference genes [10,11]. The suitable internal reference genes will be different for different cultivars, different tissues of the same cultivar, and the same tissue under different stresses. For example, in cranberries of the same family and genus, *PP2A* is the most stable internal reference gene among different cultivars [12]. In rabbiteye blueberry, *UBC28*/*RH8* is the best internal reference gene among the six organs [13]. *Actin* is the most stably expressed gene in different tissues and fruit development stages of dragon fruit [14]. *Unigene26576* and *Unigene33024* are the most suitable reference genes in *Miscanthus Sinensis* root tissue under various abiotic stresses [15]. Hence, identifying stable reference genes under species-specific treatment conditions is a prerequisite for qRT-PCR analyses.

To date, the reference genes selected for *Vaccinium* spp. species have mainly been blueberry [16] and cranberry genes [12]. There are still no systematic studies on suitable reference genes for gene expression analyses of different lingonberry cultivars and tissues. In this study, qRT-PCR was used to investigate the expression levels of putative internal reference genes in various lingonberry cultivars and tissues and in lingonberry plants exposed to two different abiotic stresses. The experimental data were statistically analyzed using GeNorm v3.5. [17], NormFinder v20. [18], and BestKeeper v1.0 [19] to assess the stability of the candidate gene expression levels. The findings of this study will enable researchers to conduct reliable gene expression analyses of lingonberry, with possible implications for the molecular breeding of new lingonberry varieties.

## 2. Results

### 2.1. RNA

Total RNA was extracted by the improved CTAB method. The lingonberry cultivar ‘Ida’, ‘Sunna’, ‘Bruch Rousi’, and ‘Red Pearl’ were collected and treated with abiotic stress using ‘Ida’ tissue culture seedlings as research materials. The extracted RNA samples were stored at −80 °C. For the quality control analysis, clear and distinct RNA bands were detected in the agarose gel, with the 28S rRNA band almost twice as bright as the 18S rRNA band. Moreover, there was no degradation or visible DNA contaminants (Figure S1).

### 2.2. Screening Results for the Candidate Internal Reference Genes

Ten candidate gene families were initially screened on the basis of the annotated library derived from the sequenced lingonberry fruit transcriptome (*Actin*, *Tub*, Tbp, *Cyp*, *Chy*, *GAPDH*, *18S rRNA*, *EF-1α*, *EF-2α*, and *EIF*) [20]. Their expression levels were determined by analyzing three cDNA libraries (green, white, and red lingonberry fruits) and then compared to reveal any differences. The 10 candidate gene families were screened to identify several non-significantly differentially expressed genes (Table S1). From the FPKM values of red fruit, green fruit, and white fruit, the gene sequences with FPKM extreme differences among <3 were selected as candidate references. The results are summarized in Table 1.

### 2.3. Gene-Specific PCR Amplification Efficiency Analysis

The qRT-PCR analysis revealed that specific amplified products were generated for all 21 putative reference genes. The single bands corresponding to the products of the qRT-PCR amplification were the expected sizes.

The single bands for 21 of these candidate internal reference genes corresponded to fragments that were approximately 80 bp long, which was in accordance with the predicted product length. Thus, the primers specific for these 21 genes were used for the subsequent analysis (Figure S2).

### 2.4. Analysis of Expression Stability

#### 2.4.1. Analysis of Ct Values

**Figure 1:**
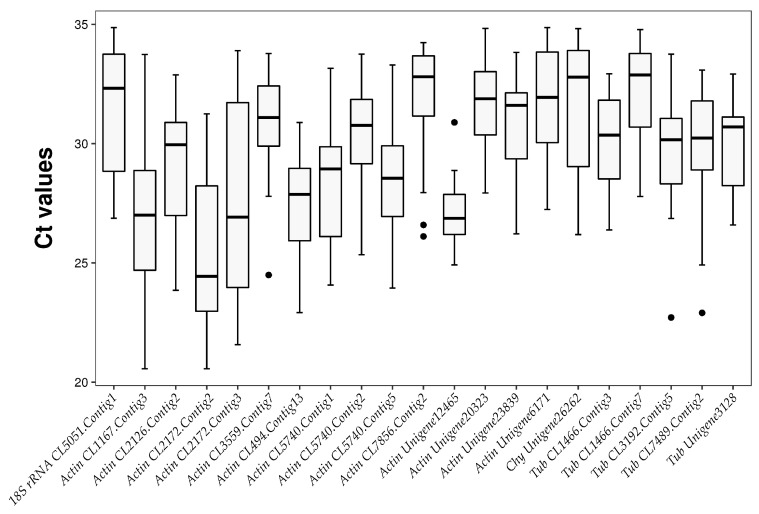
Boxplot based on cycle threshold (Ct) of 21 candidate internal reference genes in all lingonberry samples. Note: The horizontal lines from top to bottom of each box indicate maximum, upper quartile, median, lower quartile, and minimum, respectively, and the black spots outside the box indicate outliers.

The same amount of total RNA was reverse transcribed for each sample. Hence, we assumed that the range of Ct (cycle threshold) values could be used to evaluate the stability of the candidate gene expression levels. By using the mean Ct value of each experimental sample to draw a box plot (Figure 1), we quickly and intuitively examined the expression levels of these candidate genes [21]. In this way, we initially assessed how stably the 10 candidate internal reference genes were expressed in all samples under all experimental conditions (different tissues, different cultivars, and different stress treatments). All sample sets are listed in Table S2.

The Ct values of the candidate reference genes varied from 20.56 to 34.83 under all experimental conditions (Figure 1). Each candidate reference gene had a broad expression range, indicating that none of them were stably expressed across all of the lingonberry samples. According to Table S2, the most abundantly expressed gene was *Actin CL2172.Contig2*, which had the lowest average Ct ± SD (23.29 ± 0.97). Conversely, *Tub CL1466.Contig7* had the highest average Ct ± SD (32.29 ± 1.06), making it the gene with the lowest expression level. The standard deviations of *Tub CL1466.Contig7* were the smallest, indicative of the smallest variation among the examined candidate internal reference genes. Regarding the coefficient of variation (CV) of the Ct values, a candidate gene with a relatively low CV was considered to be stably expressed. The lowest and highest CVs were calculated for *Actin Unigene12465* (CV = 3.28%) and *Tub CL7489.Contig2* (CV = 7.36%), respectively.

However, assessing the stability of putative internal reference gene expression by comparing average Ct values is insufficient. Thus, we used three statistical algorithms to further analyze the expression data.

#### 2.4.2. GeNorm Analysis

The GeNorm v3.5 software uses the Q value, which is calculated using 2^−ΔCt^ (ΔCt = Ct_min_ − Ct_sample_), to determine the M value [17]. The M value is obtained through a logarithmic transformation of the pairwise ratio between the expression levels of a single candidate internal reference gene and the other candidate internal reference genes along with the calculated average standard deviation. The M value is used to rank the stability of the candidate reference genes. If a candidate reference gene has an M value less than 1.5, it is stably expressed and can be used as an alternative reference gene. Notably, the M value is negatively correlated with the stability of candidate internal reference gene expression. Furthermore, GeNorm v3.5 can be used to perform a paired difference analysis (V*n/n* + 1) of the candidate internal reference genes, thereby helping to determine the optimal number of internal reference genes. Specifically, a threshold of 0.15 is set for V*n/n* + 1. If the V*n/n* + 1 value for the test sample set exceeds 0.15, the inclusion of an additional gene (*n* + 1) as an internal reference gene will significantly enhance the expression stability. Conversely, if V*n/n* + 1 is less than 0.15, the addition of another internal reference gene is unnecessary. In cases where none of the V*n/n* + 1 values for the test sample set meet the threshold, the GeNorm v3.5 manual recommends the selection of two or three stably expressed internal reference genes on the basis of the trend in the V values.

**Figure 2:**
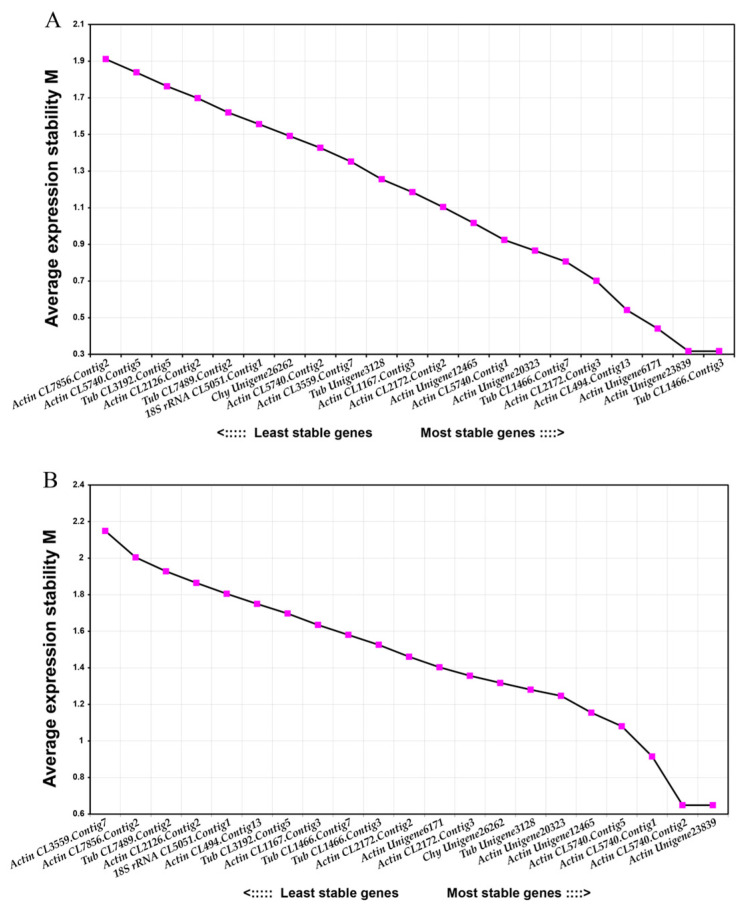
The average expression stability value (M) of the candidate reference genes in the two sample sets obtained by GeNorm v3.5 (**A**) The expression stability of candidate internal reference genes in different cultivars of lingonberry. (**B**) The expression stability of candidate internal reference genes in different tissues of lingonberry.

In the two sample sets of different lingonberry cultivars (Figure 2A), the most stably expressed candidate internal reference genes (with the lowest M values) were *Actin Unigene23839* and *Tub CL1466.Contig3*, whereas the most unstably expressed candidate internal reference gene (with the highest M value of 1.91) was *Actin CL7856.Contig2*. As shown in Figure 2A, the pairwise variation analysis showed that only two reference genes were required for the data normalization of each sample set. The V6/7 values of all sample sets were below the recognized threshold (i.e., 0.15). Therefore, *Actin Unigene23839* and *Tub CL1466.Contig3* was the best combination of internal reference genes for analyzing different lingonberry cultivars.

Figure 2B shows the M value statistics for the candidate internal reference genes in different lingonberry tissues. The most stably expressed candidate internal reference genes (with the lowest M values) were *Actin CL5740.Contig2* and *Actin Unigene23839*, whereas the most unstably expressed candidate internal reference gene (with the highest M value) was *Actin CL3559.Contig7*. According to the results of the pairwise variation analysis (Figure 2B), only two reference genes were required to normalize the data for each sample set. The V7/8 values of all sample sets were below 0.15. Thus, *Actin CL5740.Contig2* and *Actin Unigene23839* was the best combination of internal reference genes for examining different lingonberry tissues.

**Figure 3:**
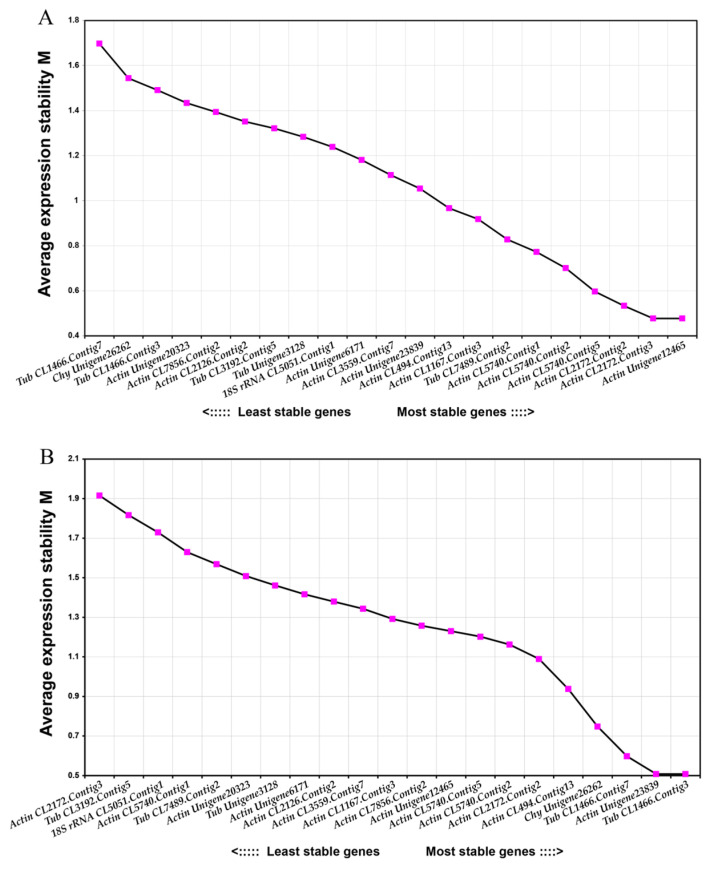
The average expression stability value (M) of the candidate reference genes in the two sample sets obtained by GeNorm v3.5. (**A**) The expression stability of candidate internal reference genes of lingonberries treated by alkali stress. (**B**) The expression stability of candidate internal reference genes of lingonberries treated by PEG-simulated drought stress.

Figure 3A provides the M value statistics for the candidate internal reference genes in the lingonberry samples exposed to alkali stress. The most stably expressed candidate internal reference genes (with the lowest M values) were *Actin CL2172.Contig3* and *Actin Unigene12465*, whereas the most unstably expressed candidate internal reference gene (with the highest M value) was *Tub CL1466.Contig7*. As shown in Figure 3A, the pairwise variation analysis showed that three reference genes were required for the data normalization of each sample set. The V3/4 values of all sample sets were below 0.15. Hence, *Actin CL2172.Contig3*, *Actin Unigene12465*, and *Actin CL2172.Contig2* was the best combination of internal reference genes for investigating lingonberry plants under alkali stress conditions.

**Figure 4:**
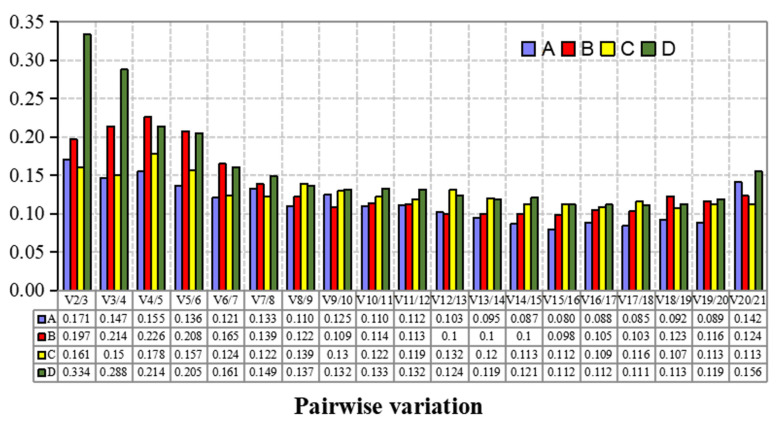
Pairwise variation (V*n*/V*n* + 1) values calculated by GeNorm v3.5. (**A**) Lingonberry treated by alkali stress; (**B**) lingonberry treated by drought stress; (**C**) lingonberry of different cultivars; (**D**) different tissues of lingonberry. Note: V*n*/V*n* + 1 > 0.15 means that an additional (*n* + 1) reference was required, whereas V*n*/V*n* + 1 ≤ 0.15 means that only *n* reference was required.

Figure 3B presents the M value statistics for the candidate internal reference genes in the lingonberry samples under simulated drought conditions. The most stably expressed candidate internal reference genes (with the lowest M values) were *Actin Unigene23839* and *Tub CL1466.Contig3*, whereas the most unstably expressed candidate internal reference gene (with the highest M value) was *Actin CL2172.Contig3*. The pairwise variation analysis (Figure 4) indicated that two reference genes were required for normalizing the data in each sample set. The V7/8 values of all sample sets were below 0.15. Accordingly, for analyzing lingonberry samples exposed to simulated drought stress, *18S rRNA CL5051.Contig1* was the best choice as a single internal reference gene, whereas *Actin Unigene23839* and *Tub CL1466.Contig3* was the best combination of internal reference genes (Figure 4).

#### 2.4.3. NormFinder Analysis

NormFinder v20. software calculates a stability value (S) based on the Q value (calculated from 2^−ΔCt^), combining the variance between groups. We ranked the expression stability of all candidate internal reference genes in different sample sets; the gene with the lowest S value was considered to have the most stable expression level. The S values and rankings for the candidate internal reference genes in different lingonberry cultivars are summarized in Table 2. As shown in Table 2, the most stable gene was *Actin Unigene23839*.

The S values and rankings for the candidate internal reference genes in different lingonberry tissues are summarized in Table 3. As shown in Table 3, the most stable gene was *Actin Unigene12465.*

The S values and rankings for the candidate internal reference genes in the lingonberry samples under alkali stress conditions are listed in Table 4, with specific information provided in Table 4. As shown in Table 4, *Actin CL5740.Contig2* was the most stable gene.

The S values and rankings for the candidate internal reference genes in the lingonberry samples exposed to simulated drought stress are summarized in Table 5, with specific details listed in Table 5. As shown in Table 5, the most stable gene was *Actin CL2172.Contig2*.

#### 2.4.4. BestKeeper Analysis

BestKeeper-v1.0 software evaluates expression stability using the Ct values of the internal reference genes. Our analysis of the candidate genes using BestKeeper-v1.0 indicated that *18S rRNA CL5051.Contig1* and *Tub CL3192.Contig5* were highly stable and appropriate for the data normalization in two sample sets (different cultivars and tissues) (Table 2 and Table 3). In the two sample sets (Table 4 and Table 5) comprising the leaves and roots treated with two abiotic stresses, *Actin CL3559.Contig7* and *Tub CL1466.Contig7* were the most stably expressed genes, respectively.

#### 2.4.5. Comprehensive Analysis of the Data

After comparing all of the data (Table 2, Table 3, Table 4 and Table 5), similar rankings of the expression stability under different experimental conditions were obtained for the candidate internal reference genes with all three statistical algorithms. Finally, we used the geometric mean of the rankings obtained using the three statistical algorithms to calculate the consensus ranking for all candidate reference genes (Table 2, Table 3, Table 4 and Table 5). For the sample sets consisting of different cultivars and different tissues, *Actin Unigene23839* and *Actin CL5740.Contig2* or *Actin Unigene12465* were selected as the single internal reference gene, respectively. For the sample sets comprising different cultivars and different tissues, *Actin Unigene23839* and *Tub CL1466.Contig3*, as well as *Actin CL5740.Contig2* and *Actin Unigene23839*, were selected as the optimal internal reference gene combinations. *Actin CL5740.Contig1* was the best choice as a single endogenous gene, whereas *Actin CL2172.Contig3*, *Actin Unigene12465*, and *Actin CL2172.Contig2* was the best endogenous gene combination for analyzing lingonberry subjected to abiotic stress. Furthermore, the *18S rRNA CL5051.Contig1* is the most suitable reference gene for PEG-simulated drought conditions, while *Actin Unigene23839* and *Tub CL1466.Contig3* was the best combination of internal reference genes for lingonberry under simulated drought stress conditions.

## 3. Discussion

Analyses of gene expression under different experimental conditions are important for determining gene functions. The advantages of qRT-PCR technology for analyzing gene expression include high specificity, high sensitivity, good reproducibility, good efficiency, and high accuracy. However, qRT-PCR requires a stably expressed gene as an internal reference control to ensure that accurate gene expression data are generated. There are currently no known reference genes that are stably expressed under all experimental conditions. Therefore, screening internal reference genes for specific experimental conditions is essential for optimizing the utility of qRT-PCR data.

Multiple studies have shown that combining transcriptome sequencing results with quantitative analyses can quickly identify suitable internal reference genes [14]. Most of the customized reference genes identified using transcriptome data are reportedly more stably expressed than traditional reference genes [22]. Previous studies combined transcriptome data to screen for reference genes in non-model plants, including *Moringa oleifera* Lam. [23] and *Sedum* sp. [24]. Transcriptome data can be used as auxiliary information for identifying common reference genes. In the current study, only four gene families were screened out of ten candidate gene families using the lingonberry transcriptome data. This effectively narrowed the scope of the internal reference gene screening, thereby improving the efficiency of the experiment.

In plant qRT-PCR analyses, some genes with relatively stable expression levels have been selected as candidate genes, such as *Tub*, *GAPDH*, *Actin*, and *18S rRNA* [25]. Housekeeping genes are widely present in all eukaryotic cells and participate in basic cell metabolism. They are considered to be stable in cells and under different physiological conditions [26]. *Actin* was revealed to be a stably expressed reference gene in different *Camellia sinensis* organs [27]. Similarly, its expression is reportedly stable in different tissues and organs of *Actinidia deliciosa* [28]. However, an increasing number of studies suggest that traditional reference genes may be affected by different experimental conditions [29]. *Actin* is stably expressed in *Arabidopsis thaliana* under abiotic stress conditions (salt, drought, and cold) [30,31,32,33,34], in tobacco exposed to abiotic stress (heat, cold, drought, and salt) [35], and in rice treated with NaCl and ABA [36]. However, *Actin* is unstably expressed in blueberry under abiotic stress conditions [16]. In the present study, *Actin* expression was most stable in lingonberry samples treated with alkali stress.

Four related genes were detected by qRT-PCR (i.e., *18S rRNA*, *Actin*, *Chy*, and *Tub*) in this study. These candidate genes were analyzed using different algorithms under different experimental conditions. *Actin 23839* was the best reference gene for different cultivars, whereas *Actin 5740/12465* was the most suitable reference gene for different tissuess. Additionally, *18S rRNA* was appropriate for analyzing lingonberry treated with drought stress. In cranberry, which belongs to the same family as lingonberry [12], *PP2A* was identified as the best internal reference gene for examining different cultivars. In contrast, *SAND* is the ideal internal reference gene for analyzing different organs and the effects of drought stress. These findings represent additional evidence of the importance of screening for suitable internal reference genes under specific experimental conditions.

Several factors may be responsible for the above-mentioned variations and observed differences in the expression stability of candidate reference genes. First, RNA expression levels are not constant under all conditions, with those of internal reference genes varying because of differences in factors, including the cell cycle stage, species, materials, and sequencing libraries. Second, we used three main algorithms (NormFinder v20., GeNorm v3.5 and BestKeeper v1.0) to analyze the study data [37,38,39]. These three algorithms are currently used by researchers to assess the stability of candidate internal reference genes for qRT-PCR analyses. NormFinder v20. can be used to identify the best reference gene or best combination, whereas GeNorm v3.5 is useful for selecting a variety of reference genes and then ranking them according to suitability. Unlike NormFinder v20. and GeNorm v3.5, BestKeeper-1.0 does not require the preprocessing of data; it can directly use Ct values obtained by qRT-PCR for calculations [39]. A comprehensive analysis using multiple methods is the best way to screen for the optimal reference gene.

Although there may be some inconsistencies between our results and the findings of earlier studies, our observations indicate that the reference gene most suitable for a set of experimental conditions and a specific analysis should be selected and further evaluated prior to analyzing gene expression levels.

## 4. Materials and Methods

### 4.1. Plant Materials, Treatment, and Tissue Collection

The lingonberry cultivar ‘Ida’, ‘Sunna’, ‘Bruch Rousi’ and ‘Red Pearl’ were used during the 2017 season in this study. We collected the fresh, tender parts of ‘Ida’ from roots, stems, leaves, leaf buds and flower buds. Flowers were gathered at the full-bloom phase, and green, white and red fruits respectively collected 20, 50 and 80 days after flowering. The tissue culture seedling of “Ida” was used as the research materials of abiotic stress treatment. The roots and leaves were collected after stress treatment.

All samples were collected from plants grown at the Engineering Center of Genetic Breeding and Innovative Utilization of Small Fruits of Jilin Province, Changchun, China (125°42′ N, 43°80′ E). Lingonberry (Ida) seedling cuttings were used for the abiotic stress treatments, after which the roots and leaves were collected. All samples were immediately frozen in liquid nitrogen and stored at −80 °C before being analyzed in follow-up experiments.

Lingonberry (Ida) seedling cuttings were rooted in rooting medium for the abiotic stress treatments. Consistently growing seedlings with a well-developed root system were selected from the group culture. Then, the medium on the seedlings was cleaned and put the roots of the seedlings in the culture solution placed in a 5 × 5 sponge plate, which was set in a 5 L mineral water bottle without the upper half and containing 1 L abiotic stress culture solution. The plants were grown in a controlled climate chamber (23 °C, 16-h/8-h photoperiod and 50% relative humidity). Using a completely randomized experimental design, uniformly growing healthy lingonberry seedlings were exposed to alkali stress or drought stress. For the alkali stress treatment, the plants were cultured in 5 L Hoagland’s nutrient solution (pH 7.6) containing 100 mmol L^−1^ NaHCO_3_. For the PEG-simulated drought stress treatment, the nutrient solution was supplemented with 8% PEG 8000. The untreated seedlings were cultured in 4 L Hoagland nutrient solution (pH 4.5–5.0) as a control. Leaf and root samples were collected at four-time points (0, 4, 8, and 12 h), with three biological replicates each.

### 4.2. RNA Isolation and cDNA Synthesis

Total RNA was extracted using a modified CTAB method [40]. The quantity and quality of the extracted RNA were determined using the Implen P330 (IMPLEN, Munich, Germany) instrument. Only RNA samples satisfying the following criteria were used: (1) absorbance ratios of A260/A280 = 1.8–2.1 and A260/A230 = approximately 2.0; (2) clear and distinct (i.e., no smearing) 28S/18S ribosomal RNA bands in 1.2% (*w*/*v*) agarose gels. To ensure the consistency of each reaction, cDNA was synthesized in 20 μL solutions containing 1000 ng template RNA using the PrimeScript RT reagent kit with gDNA Eraser thorugh a two-step process (Perfect Real Time) (Takara, Dalian, China). All cDNA samples were stored at −20 °C.

### 4.3. Selection of Candidate Reference Genes

We sequenced the transcriptome and created annotation and cDNA libraries for the green, white, and red fruits, which corresponded to three lingonberry fruit development stages (NCBI SAMN07305417, SAMN07305418 and SAMN07305419). Some Unigenes were identified for each gene family (*Actin*, *Tub*, *Tbp*, *Cyp*, *Chy*, *GAPDH*, *18S rRNA*, EF-1α, *EF-2α*, and *EIF*). The expression levels of all the initially screened genes were calculated through the FPKM (Fragments Per Kilobase Million) method using the formula: FPKM = (10^9^) × C/N × L. FPKM is the expression level of tested Unigene X, C is the number of Unigene X reads that are aligned, N is the total number of reads of all Unigenes that are aligned, and L is the total number of bases of Unigene X. A |log_2_Ratio| ≥ 1 indicated a significant expression difference between the libraries. Finally, we selected the gene sequence in each of the 10 candidate reference gene families that had the smallest |log_2_Ratio| value (<1) [41,42].The quantitative relationship of differential expression values was determined using the formula log_2_(R_FPKM/G_FPKM), log_2_(W_FPKM/G_FPKM), and log_2_(R_FPKM/W_FPKM), where R_FPKM, W_FPKM, and G_FPKM is the expression level of the gene in red, white, and green fruit. Genes satisfying the following criterion were considered as candidate reference genes: |log_2_ ratio| < 2 [43,44,45,46]. The results showed that there was no significant difference in expression between red fruit, white fruit and green fruit. Then, from the FPKM values of green fruit, white fruit and red fruit, the gene sequences with extreme differences among <3 were selected as candidate reference genes and primers were designed (Table S1).

### 4.4. Primer Design and Analysis of the Amplification Efficiency for qRT-PCR

The Primer 3.0 online tool (http://bioinfo.ut.ee/primer3/, accessed on 7 May 2019) was used to design gene-specific qRT-PCR primers for 21 candidate reference genes. All qRT-PCR primers were synthesized by Suzhou Genewiz Bio-Technology Services Co. (Suzhou, China): Tm values are 58–62 °C, GC contents are 45–55%, primer lengths are 18–25 bp, and product lengths are 80–150 bp. Using a 4-fold serially diluted template yielded amplification products with high efficiency and specificity. A primer of 0.4 μ was used for fluorescence quantitative PCR reaction Amplification efficiencies of candidate reference gene primers varied between 90.482~104.69%.

The BLAST online tool (http://blast.ncbi.nlm.nih.gov/Blast.CGI, accessed on 16 June 2019) was used to analyze primer specificity [46]. The PCR amplification program was as follows: a. Predenaturation: 94 °C, 5 min; b. Denaturation: 94 °C, 40 s; c. Annealing: Tm (Tm value is each candidate internal reference gene primer Tm), 40 s; d. Extension: 72 °C, 40 s, a total of 35 cycles; e. Final extension: 72 °C, 10 min. The amplified products were examined by 2.5% (*w*/*v*) agarose gel electrophoresis to verify primer specificity.

The qRT-PCR analysis was performed using the SYBR Green PCR Master Mix system (TaKaRa, Dalian, China) and the Applied Biosystems StepOne Plus Realtime PCR System (Thermo Fisher, Waltham, MA, USA) as previously described [47], with three biological replicates prepared using independently isolated RNA. Relative gene expression levels were calculated using the 2^−ΔCt^ method (Table S3) [48].

### 4.5. Determination and Validation of Reference Gene Expression Stability

The average Ct value for the three biological replicates was calculated. In addition, the gene expression range of variation was calculated using the formula ΔCt = Ct_max_ − Ct_min_. To assess the utility of the candidate reference genes, the generated data were statistically analyzed using GeNorm v3.5. [17], NormFinder v20. [18], and BestKeeper v1.0 [19]. These three methods are the ones currently used by researchers to assess the stability of candidate genes for use as reference genes in qRT-PCR analyses. NormFinder v20. can generate the best reference gene or best combination, whereas GeNorm v3.5 can select a combination of reference genes and rank them by suitability. Unlike NormFinder v20. and GeNorm v3.5., BestKeeper 1.0 does not require preprocessing of data and can directly make use of Ct values obtained by qRT-PCR for calculations.

## 5. Conclusions

In this study, 21 candidate reference genes were selected on the basis of transcriptome sequencing results and published information. Their expression stability under different conditions was evaluated using three algorithms. *Actin* was revealed as the best internal reference gene when all two abiotic stresses were considered simultaneously; however, when the leaves and roots were treated separately with the three abiotic stresses, the *18s rRNA* and *Actin*/*Tub* genes were identified as the most appropriate internal reference genes, respectively. Both *Actin* and *Tub* were suitable internal reference genes for analyzing the different lingonberry cultivars. In different tissues, *Actin* was the most stably expressed gene. These reference genes will be useful for future studies on the molecular mechanisms underlying stress resistance and for the breeding of novel lingonberry varieties.

## Figures and Tables

**Table 1:** Genes and primer sequences for primer design in 10 candidate gene families.

A	Gene ID	Primer Sequences	Amplicfication Efficency (%)	Amplicon Length (bp)	TM (°C)
*Actin*	*CL1167.Contig3_All*	F: GCCAAATCATCGCCGTGTT	92.482	80	82.56
		R: CCTCTCCTGTCACTGCTTTAATCTC			
*Actin*	*CL2126.Contig2_All*	F: GCCTTCAACCAACCAGACTTACG	97.940	82	85.21
		R: GAACTAGAATCCCAGAGGCAAATG			
*Actin*	*CL2172.Contig2_All*	F: CCGACTGAAATGGATCTCGAA	97.220	80	83.27
		R: GCGATGCGAAAACCCTCTATAC			
*Actin*	*CL2172.Contig3_All*	F: CCGACTGAAATGGATCTCGAA	104.69	80	81.86
		R: GCGATGCGAAAACCCTCTATAC			
*Actin*	*CL3559.Contig7_All*	F: CAGAAGCGCCTCTCAATCCA	90.482	82	79.24
		R: CATAGCAGGAGCGTTGAACGT			
*Actin*	*CL494.Contig13_All*	F: GTCGGCTCTAAATCCAGAATCCT	95.123	80	82.63
		R: TTCGGAGAGAAGCTGAGAAGCA			
*Actin*	*CL5740.Contig1_All*	F: ACCTTTTGGATTGTGGGCTAGA	91.052	80	83.56
		R: AGCTCCACTTGCACTTTTCCTT			
*Actin*	*CL5740.Contig2_All*	F: ACCTTTTGGATTGTGGGCTAGA	98.549	81	80.23
		R: AGCTCCACTTGCACTTTTCCTT			
*Actin*	*CL5740.Contig5_All*	F: CTGGACAAAAGGCCGGAATT	94.082	80	82.64
		R: TTTGCCATGTGCAGACTTTGG			
*Actin*	*CL7856.Contig2_All*	F: GCTCCTGCTTGCCTTCTTGT	98.792	80	82.36
		R: CCCTGATAGCAGGATCTCAAGTTT			
*Actin*	*Unigene12465_All*	F: CTGGTAGCAAAACCCCACTCTGA	96.517	80	83.49
		R: ATCCCACCTCCTTGGCCATAT			
*Actin*	*Unigene20323_All*	F: TCGCAGCCTCAACTCCAAAT	95.378	80	84.91
		R: CCATTAACGGTGGCAAATCTC			
*Actin*	*Unigene23839_All*	F: TACTGACACTGCCCTTTGCTTTG	95.836	80	78.66
		R: ACTTGCGACCAAGCATTTCC			
*Actin*	*Unigene6171_All*	F: ACGCCTGGGAAAAGACAAAA	94.019	80	81.84
		R: AGAACCGACGACACCATTGAC			
*Chy*	*Unigene26262_All*	F: CATTGTGATGGCTGCGGTAT	103.26	83	82.36
		R: GGCCTAAGCTAATCGAGATGCTT			
*18S rRNA*	*CL5051.Contig1_All*	F: CAACCTCTCCCGCCAAATCT	96.502	80	85.42
		R: GCAGTGGTGGTGATGCCATT			
*Tub*	*CL1466.Contig3_All*	F: ACGTCCAAGGTGGCCAATGT	96.525	80	84.25
		R: TGGGTCTATGCCGTGTTCATC			
*Tub*	*CL1466.Contig7_All*	F: ACTCAGCACCCCATCCTTTG	97.366	80	79.25
		R: GGAATCGCAAGCAGCAAGTC			
*Tub*	*CL3192.Contig5_All*	F: TTGGACCGCATTCGTAAGC	99.78	80	83.45
		R: GTACCCCCACCAACAGCATT			
*Tub*	*CL7489.Contig2_All*	F: ATCGACCTTGCAGGCCTGTT	100.677	81	81.32
		R: CTCCCGACAAGCTTCGGATATC			
*Tub*	*Unigene3128_All*	F: CGGAAGCGATTTACTGAGGAA	100.745	80	80.54
		R: TGTATGTTGTGCCGCTCACA			

**Table 2:** The stability ranking of 21 candidate internal reference genes in different cultivars of lingonberry calculated by GeNorm v3.5, NormFinder v20., and BestKeeper-v1.0. software.

Gene	Different Cultivars Samples in Lingonberry							
	GeNorm		NormFinder		BestKeeper			Com.
	M	Rank	S	Rank	SD	CV (%)	Rank	Rank
*18S rRNA CL5051.Contig1*	1.56	16	1.262	16	0.44	1.55	1	11
*Actin CL1167.Contig3*	1.18	11	0.804	9	1.21	4.45	7	9
*Actin CL2126.Contig2*	1.70	18	1.434	18	1.49	5.32	10	15
*Actin CL2172.Contig2*	1.10	10	0.871	11	1.42	6.31	8	10
*Actin CL2172.Contig3*	0.70	5	0.532	6	1.10	4.67	4	2
*Actin CL3559.Contig7*	1.35	13	1.101	13	1.59	5.75	13	14
*Actin CL494.Contig13*	0.54	4	0.449	4	1.80	6.57	15	8
*Actin CL5740.Contig1*	0.92	8	0.641	8	2.05	7.52	17	11
*Actin CL5740.Contig2*	1.43	14	1.125	14	2.29	7.79	18	15
*Actin CL5740.Contig5*	1.84	20	1.594	20	2.92	10.48	20	21
*Actin CL7856.Contig2*	1.91	21	1.630	21	1.10	3.44	4	15
*Actin Unigene12465*	1.02	9	0.852	10	0.54	2.04	2	7
*Actin Unigene20323*	0.87	7	0.469	5	1.19	3.87	6	6
*Actin Unigene23839*	0.32	1	0.110	1	1.54	5.20	12	1
*Actin Unigene6171*	0.44	3	0.319	3	1.50	5.16	11	4
*Chy Unigene26262*	1.49	15	1.201	15	2.00	6.48	16	15
*Tub CL1466.Contig3*	0.32	1	0.309	2	1.61	5.54	14	4
*Tub CL1466.Contig7*	0.81	6	0.586	7	1.02	3.22	3	3
*Tub CL3192.Contig5*	1.76	19	1.448	19	2.32	8.48	19	20
*Tub CL7489.Contig2*	1.62	17	1.293	17	3.47	12.69	21	19
*Tub Unigene3128*	1.23	12	1.006	12	1.43	5.09	9	11
Best genes	*Actin Unigene23839/*		*Actin Unigene23839*		*18S rRNA CL5051.Contig1*			*Actin Unigene 23839*
	*Tub CL1466.Contig3*							
Worst genes	*Actin CL7856.Contig2*		*Actin CL7856.Contig2*		*Tub CL7489.Contig2*			*Actin CL5740 Contig5*

**Table 3:** The stability ranking of 21 candidate internal reference genes in different tissues of lingonberry using the GeNorm v3.5, NormFinder v20., and BestKeeperv1.0 software.

Gene	Different Tissues Samples in Lingonberry							
	GeNorm		NormFinder		BestKeeper			Com.
	M	Rank	S	Rank	SD	CV (%)	Rank	Rank
*18S rRNA CL5051.Contig1*	1.80	17	1.341	17	1.44	4.95	10	16
*Actin CL1167.Contig3*	1.63	14	1.162	14	1.73	6.41	15	15
*Actin CL2126.Contig2*	1.86	18	1.414	18	1.90	6.88	18	18
*Actin CL2172.Contig2*	1.46	11	0.779	9	1.09	4.89	4	8
*Actin CL2172.Contig3*	1.36	9	0.658	7	0.91	3.90	3	5
*Actin CL3559.Contig7*	2.15	21	2.253	21	1.63	5.18	12	18
*Actin CL494.Contig13*	1.75	16	1.176	15	1.37	5.34	8	14
*Actin CL5740.Contig1*	0.91	3	0.615	3	1.68	6.34	14	7
*Actin CL5740.Contig2*	0.65	1	0.567	2	1.24	4.32	5	1
*Actin CL5740.Contig5*	1.08	4	0.624	4	1.38	5.21	9	3
*Actin CL7856.Contig2*	2.00	20	1.657	20	2.71	9.02	21	21
*Actin Unigene12465*	1.15	5	0.514	1	0.86	3.19	2	1
*Actin Unigene20323*	1.25	6	0.642	6	1.31	4.33	6	4
*Actin Unigene23839*	0.65	1	0.636	5	1.65	5.68	13	5
*Actin Unigene6171*	1.40	10	0.964	10	1.80	5.94	17	13
*Chy Unigene26262*	1.32	8	1.003	11	1.75	6.01	16	12
*Tub CL1466.Contig3*	1.53	12	1.091	13	1.93	6.63	19	16
*Tub CL1466.Contig7*	1.58	13	1.073	12	1.31	4.26	6	10
*Tub CL3192.Contig5*	1.70	15	1.191	16	0.82	2.69	1	11
*Tub CL7489.Contig2*	1.93	19	1.524	19	2.38	8.21	20	20
*Tub Unigene3128*	1.28	7	0.712	8	1.53	5.18	11	9
Best genes	*Actin CL5740.Contig2/*		*Actin Unigene12465*		*Tub CL3192.Contig5*			*Actin CL5740.Contig2/*
	*Actin Unigene23839*							*Actin Unigene 12465*
Worst genes	*Actin CL3559.Contig7*		*Actin CL3559.Contig7*		*Actin CL7856.Contig2*			*Tub CL7489.Contig2*

**Table 4:** The stability ranking of 21 candidate internal reference genes in lingonberry treated by alkali stress using the GeNorm v3.5, NormFinder v20., and BestKeeperv1.0 software.

Gene	Lingonberries Treated by Alkali Stress							
	GeNorm		NormFinder		BestKeeper			Com.
	M	Rank	S	Rank	SD	CV (%)	Rank	Rank
*18S rRNA CL5051.Contig1*	1.24	13	0.942	16	1.62	5.28	21	18
*Actin CL1167.Contig3*	0.92	8	0.847	15	1.55	5.08	18	14
*Actin CL2126.Contig2*	1.35	16	0.842	13	1.25	4.08	15	16
*Actin CL2172.Contig2*	0.53	3	0.551	4	0.79	3.24	6	2
*Actin CL2172.Contig3*	0.48	1	0.642	7	1.16	4.46	13	5
*Actin CL3559.Contig7*	1.11	11	0.745	9	0.45	1.40	1	5
*Actin CL494.Contig13*	0.97	9	0.535	3	1.16	4.03	13	10
*Actin CL5740.Contig1*	0.77	6	0.361	2	0.60	1.98	3	1
*Actin CL5740.Contig2*	0.70	5	0.359	1	0.95	2.98	8	3
*Actin CL5740.Contig5*	0.60	4	0.634	6	1.02	3.37	10	4
*Actin CL7856.Contig2*	1.39	17	0.945	17	0.51	1.53	2	13
*Actin Unigene12465*	0.48	1	0.779	11	1.15	4.16	12	9
*Actin Unigene20323*	1.43	18	1.072	18	0.62	1.88	5	14
*Actin Unigene23839*	1.05	10	0.560	5	0.94	2.89	7	7
*Actin Unigene6171*	1.18	12	0.657	8	0.60	1.79	3	8
*Chy Unigene26262*	1.54	20	1.236	20	1.55	4.69	18	20
*Tub CL1466.Contig3*	1.49	19	1.141	19	1.47	4.80	17	19
*Tub CL1466.Contig7*	1.70	21	2.058	21	1.61	4.93	20	21
*Tub CL3192.Contig5*	1.32	15	0.843	14	1.33	4.45	16	17
*Tub CL7489.Contig2*	0.83	7	0.753	10	1.10	3.59	11	11
*Tub Unigene3128*	1.28	14	0.808	12	0.95	3.08	8	12
Best genes	*Actin CL2172.Contig3/*		*Actin CL5740.Contig2*		*Actin CL3559.Contig7*			*Actin CL5740.Contig1*
	*Actin Unigene12465*							
Worst genes	*Tub CL1466.Contig7*		*Tub CL1466.Contig7*		*18S rRNA CL5051.Contig1*			*Tub CL1466.Contig7*

**Table 5:** The stability ranking of 21 candidate internal reference genes in lingonberry treated by PEG-simulated drought stress using the GeNorm v3.5, NormFinder v20., and BestKeeperv1.0 software.

Gene	Lingonberries Treated by PEG-Simulated Drought Stress							
	GeNorm		NormFinder		BestKeeper			Com.
	M	Rank	S	Rank	SD	CV (%)	Rank	Rank
*18S rRNA CL5051.Contig1*	1.73	19	1.510	19	1.96	6.14	19	19
*Actin CL1167.Contig3*	1.29	11	0.820	12	1.27	4.22	16	14
*Actin CL2126.Contig2*	1.38	13	0.649	4	0.99	3.31	8	9
*Actin CL2172.Contig2*	1.09	6	0.439	1	0.58	2.45	4	1
*Actin CL2172.Contig3*	1.91	21	1.795	21	2.44	8.79	21	21
*Actin CL3559.Contig7*	1.34	12	0.661	5	0.72	2.32	7	8
*Actin CL494.Contig13*	0.94	5	0.482	2	0.69	2.40	6	3
*Actin CL5740.Contig1*	1.63	18	1.302	18	1.61	5.45	18	18
*Actin CL5740.Contig2*	1.16	7	0.697	6	1.01	3.23	12	9
*Actin CL5740.Contig5*	1.20	8	0.764	9	1.15	3.93	13	11
*Actin CL7856.Contig2*	1.26	10	1.071	15	0.99	3.04	8	12
*Actin Unigene12465*	1.23	9	0.606	3	1.00	3.71	11	7
*Actin Unigene20323*	1.51	16	1.240	17	1.19	3.62	14	16
*Actin Unigene23839*	0.51	1	0.766	10	0.55	1.71	3	4
*Actin Unigene6171*	1.42	14	0.982	14	0.99	2.96	8	13
*Chy Unigene26262*	0.75	4	0.746	8	0.36	1.07	2	4
*Tub CL1466.Contig3*	0.51	1	0.814	11	0.65	2.10	5	6
*Tub CL1466.Contig7*	0.60	3	0.745	7	0.28	0.82	1	1
*Tub CL3192.Contig5*	1.82	20	1.523	20	2.26	7.40	20	20
*Tub CL7489.Contig2*	1.57	17	1.180	16	1.53	4.94	17	17
*Tub Unigene3128*	1.46	15	0.847	13	1.20	3.91	15	15
Best genes	*Actin Unigene23839/*		*Actin CL2172.Contig2*		*Tub CL1466.Contig7*			*18S rRNA CL5051.* *Contig1*
	*Tub CL1466.Contig3*							
Worst genes	*Actin CL2172.Contig3*		*Actin CL2172.Contig3*		*Actin CL2172.Contig3*			*Actin CL2172.Contig3*

## Data Availability

Data are contained within the article and Supplementary Materials.

## Supplementary Materials

The following supporting information can be downloaded at: https://www.mdpi.com/article/10.3390/plants12244180/s1, Figure S1: 1.2% agarose gel electrophoresis detection of total RNA extracted from lingonberry. Figure S2: Products of qRT-PCR of 21 candidate reference genes. Table S1: Non-significantly differentially expressed genes in lingonberries among 10 candidate gene families. Table S2: Average Ct value, standard deviation (SD) and coefficient of variation (CV) of candidate internal reference genes in all lingonberry samples. Table S3: The melting curves of 21 candidate reference mRNA genes.

## References

[1] Brennan R. (2012). Blueberries. By JB Retamales and JF Hancock. Wallingford, UK: CABI (2012), pp. 336, £ 45.00. ISBN 978-1-84593-826-0. Exp. Agric..

[2] Brown P.N., Turi C.E., Shipley P.R., Murch S.J. (2012). Comparisons of large (*Vaccinium macrocarpon* Ait.) and small (*Vaccinium oxycoccos* L., *Vaccinium vitis-idaea* L.) cranberry in British Columbia by phytochemical determination, antioxidant potential, and metabolomic profiling with chemometric analysis. Planta Med..

[3] Karppinen K., Zoratti L., Nguyenquynh N., Häggman H., Jaakola L. (2016). On the developmental and environmental regulation of secondary metabolism in *Vaccinium* spp. Berries. Front. Plant Sci..

[4] Vanguilder H.D., Vrana K.E., Freeman W.M., VanGuilder H.D., Vrana K.E., Freeman W.M. (2008). Twenty-five years of quantitative PCR for gene expression analysis. BioTechniques.

[5] Wang X., Fu Y., Ban L., Wang Z., Feng G., Li J., Gao H. (2014). Selection of reliable reference genes for quantitative real-time RT-PCR in alfalfa. Genes Genet. Syst..

[6] Wang L., Pan Y., Yang L., Cai S., Huang X. (2013). Validation of internal reference genes for qRT-PCR normalization in ‘Guanxi Sweet Pummelo’ *(Citrus grandis*). J. Fruit Sci..

[7] Goidin D., Mamessier A., Staquet M.J., Schmitt D., Berthier-Vergnes O. (2001). Ribosomal 18S RNA prevails over glyceraldehyde-3-phosphate dehydrogenase and beta-actin genes as internal standard for quantitative comparison of mRNA levels in invasive and noninvasive human melanoma cell subpopulations. Anal. Biochem..

[8] Wang M., Lu S. (2016). Validation of suitable reference genes for quantitative gene expression analysis in *Panax ginseng*. Front. Plant Sci..

[9] Erickson H.S., Albert P.S., Gillespie J.W., Rodriguez-Canales J., Marston Linehan W., Pinto P.A., Chuaqui R.F., Emmert-Buck M.R. (2009). Quantitative RT-PCR gene expression analysis of laser microdissected tissue samples. Nat. Protoc..

[10] Wang H.L., Chen J.H., Tian Q.Q., Xia X.L., Yin W.L. (2014). Identification and validation of reference genes for *Populus euphratica* gene expression analysis during abiotic stresses by quantitative real-time PCR. Physiol. Plant..

[11] Wang H.-L., Li L., Tang S., Yuan C., Tian Q., Su Y., Li H.-G., Zhao L., Yin W., Zhao R. (2015). Evaluation of appropriate reference genes for reverse Transcription-Quantitative PCR studies in different tissues of a desert poplar via comparision of different algorithms. Int. J. Mol. Sci..

[12] Li C., Xu J., Deng Y., Sun H., Li Y. (2019). Selection of reference genes for normalization of cranberry (*Vaccinium macrocarpon* Ait.) gene expression under different experimental conditions. PLoS ONE.

[13] Vashisth T., Johnson L.K., Malladi A. (2011). An efficient RNA isolation procedure and identification of reference genes for normalization of gene expression in blueberry. Plant Cell Rep..

[14] Chen C., Wu J., Hua Q., Tel-Zur N., Qin Y. (2019). Identification of reliable reference genes for quantitative real-time PCR normalization in pitaya. Plant Methods.

[15] Huang Y.Z., Tang M.Y., Zhong M.Y., Wang R.J., Chen J.Y., Ye Y.T., Zhang X.Q., Nie G. (2020). Optimum reference gene selection in miscanthus sinensis root tissue with various abiotic stress. J. Sichuan Agric. Univ..

[16] Deng Y., Li Y., Sun H. (2020). Selection of reference genes for RT-qPCR normalization in blueberry (*Vaccinium corymbosum* × *angustifolium*) under various abiotic stresses. FEBS Open Bio.

[17] Vandesompele J., De Preter K., Pattyn F., Poppe B., Van Roy N., De Paepe A., Speleman F. (2002). Accurate normalization of real-time quantitative RT-PCR data by geometric averaging of multiple internal control genes. Genome Biol..

[18] Andersen C.L., Jensen J.L., Ørntoft T.F. (2004). Normalization of real-time quantitative reverse transcription-PCR data: A model-based variance estimation approach to identify genes suited for normalization, applied to bladder and colon cancer data sets. Cancer Res..

[19] Pfaffl M.W., Tichopad A., Prgomet C., Neuvians T.P. (2004). Determination of stable housekeeping genes, differentially regulated target genes and sample integrity: BestKeeper--Excel-based tool using pair-wise correlations. Biotechnol. Lett..

[20] Tian Y., Ma Z., Ma H., Gu Y., Li Y., Sun H. (2020). Comparative transcriptome analysis of lingonberry (*Vaccinium vitis-idaea*) provides insights into genes associated with flavonoids metabolism during fruit development. Biotechnol. Biotechnol. Equip..

[21] Royeen C.B. (1986). The boxplot: A screening test for research data. Am. J. Occup. Ther..

[22] Yang D., Li Q., Wang G.X., Ma Q.H., Zhu L.Q. (2017). Reference genes selection and system establishment for Real-Time qPCR analysis in *Ping’ou Hybrid Hazelnut* (*C. heterophylla Fisch*. × *C. avellana* L.). Sci. Agric. Sin..

[23] Deng L.T. (2016). Transcriptome Based Reference Genes Selection and the Expression Analyses of Genes Involved in the Biosyn-Thesis of Three Metabolic Pathway. Ph.D. Thesis.

[24] Sang J. (2014). Validation of Reference Genes and Analysis of HSF Gene Family Based on RNA-Seq in Hyper-Accumulating *Se-dum alfredii* Hance. Ph.D. Thesis.

[25] Mallona I., Lischewski S., Weiss J., Hause B., Egea-Cortines M. (2010). Validation of reference genes for quantitative real-time PCR during leaf and flower development in Petunia hybrida. BMC Plant Biol..

[26] Stürzenbaum S.R., Kille P. (2001). Control genes in quantitative molecular biological techniques: The variability of invariance. Comp. Biochem. Physiol. Part B Biochem. Mol. Biol..

[27] Sun M., Wang Y., Yang D., Wei C., Gao L., Xia T., Shan Y., Luo Y. (2010). Reference genes for real-time fluorescence quantitative PCR in Camellia sinensis. Chin. Bull. Bot..

[28] Zhang J.Y., Huang S.N., Wang T., Pan D.L., Zhai M., Guo Z.R. (2018). Screening of reference genes for reverse transcription quantitative real-time PCR in Actinidida deliciosa. Acta Agric. Shanghai.

[29] Thellin O., Zorzi W., Lakaye B., Borman B.D., Coumans B., Hennen G., Grisar T., Igout A., Heinen E. (1999). Housekeeping genes as internal standards: Use and limits. J. Biotechnol..

[30] Atkinson N.J., Lilley C.J., Urwin P.E. (2013). Identification of genes involved in the response of *Arabidopsis* to simultaneous biotic and abiotic stresses. Plant Physiol..

[31] Egert A., Keller F., Peters S. (2013). Abiotic stress-induced accumulation of raffinose in *Arabidopsis* leaves is mediated by a single raffinose synthase (RS5, At5g40390). BMC Plant Biol..

[32] Gong X., Liu M., Zhang L., Ruan Y., Ding R., Ji Y., Zhang N., Zhang S., Farmer J., Wang C. (2015). *Arabidopsis* AtSUC2 and AtSUC4, encoding sucrose transporters, are required for abiotic stress tolerance in an ABA-dependent pathway. Physiol. Plant.

[33] Liu Y., Ji X., Nie X., Qu M., Zheng L., Tan Z., Zhao H., Huo L., Liu S., Zhang B. (2015). *Arabidopsis* AtbHLH112 regulates the expression of genes involved in abiotic stress tolerance by binding to their E-box and GCG-box motifs. New Phytol..

[34] Msanne J., Lin J., Stone J.M., Awada T. (2011). Characterization of abiotic stress-responsive *Arabidopsis thaliana* RD29A and RD29B genes and evaluation of transgenes. Planta.

[35] Schmidt G.W., Delaney S.K. (2010). Stable internal reference genes for normalization of real-time RT-PCR in tobacco (Nicotiana tabacum) during development and abiotic stress. Mol. Genet. Genom..

[36] Hashimoto M., Kisseleva L., Sawa S., Furukawa T., Komatsu S., Koshiba T. (2004). A novel rice PR10 protein, RSOsPR10, specifically induced in roots by biotic and abiotic stresses, possibly via the jasmonic acid signaling pathway. Plant Cell Physiol..

[37] Ashrafi M., Azimi Moqadam M.R., Moradi P., Mohsenifard E., Shekari F. (2018). Evaluation and validation of housekeeping genes in two contrast species of thyme plant to drought stress using real-time PCR. Plant Physiol. Biochem..

[38] Joseph J.T., Poolakkalody N.J., Shah J.M. (2018). Plant reference genes for development and stress response studies. J. Biosci..

[39] Tang X., Zhang N., Si H., Calderón-Urrea A. (2017). Selection and validation of reference genes for RT-qPCR analysis in potato under abiotic stress. Plant Methods.

[40] Chang S., Puryear J., Cairney J. (1993). A simple and efficient method for isolating RNA from pine trees. Plant Mol. Biol. Report..

[41] Artico S., Nardeli S.M., Brilhante O., Grossi-de-Sa M.F., Alves-Ferreira M. (2010). Identification and evaluation of new reference genes in Gossypium hirsutum for accurate normalization of real-time quantitative RT-PCR data. BMC Plant Biol..

[42] Diretto G., Welsch R., Tavazza R., Mourgues F., Pizzichini D., Beyer P., Giuliano G. (2007). Silencing of beta-carotene hydroxylase increases total carotenoid and beta-carotene levels in potato tubers. BMC Plant Biol..

[43] Libault M., Thibivilliers S., Bilgin D., Radwan O., Benitez M., Clough S., Stacey G. (2008). Identification of four soybean reference genes for gene expression normalization. The Plant Genome.

[44] Thellin O., ElMoualij B., Heinen E., Zorzi W. (2009). A decade of improvements in quantification of gene expression and internal standard selection. Biotechnol. Adv..

[45] Mortazavi A., Williams B.A., McCue K., Schaeffer L., Wold B. (2008). Mapping and quantifying mammalian transcriptomes by RNA-Seq. Nat. Methods.

[46] Altschul S.F., Gish W., Miller W., Myers E.W., Lipman D.J. (1990). Basic local alignment search tool. J. Mol. Biol..

[47] Huang X., Yang L., Jin Y., Lin J., Liu F. (2017). Generation, annotation, and analysis of a large-scale expressed sequence tag library from *Arabidopsis pumila* to explore salt-responsive genes. Front. Plant Sci..

[48] Livak K.J., Schmittgen T.D. (2001). Analysis of relative gene expression data using real-time quantitative PCR and the 2(-Delta Delta C(T)) Method. Methods.

