# Supplementary material for: Transcriptome-Based Identification of the Optimal Reference Genes for Quantitative Real-Time Polymerase Chain Reaction Analyses of Lingonberry Fruits throughout the Growth Cycle
Source: Plants (Basel). 2023 Dec 16;12(24):4180. doi: 10.3390/plants12244180 (PMC10748091; doi:10.3390/plants12244180)
Supplement: Supplementary file 1 [file plants-12-04180-s001.zip › Figures S1 and S2-plants.pdf]

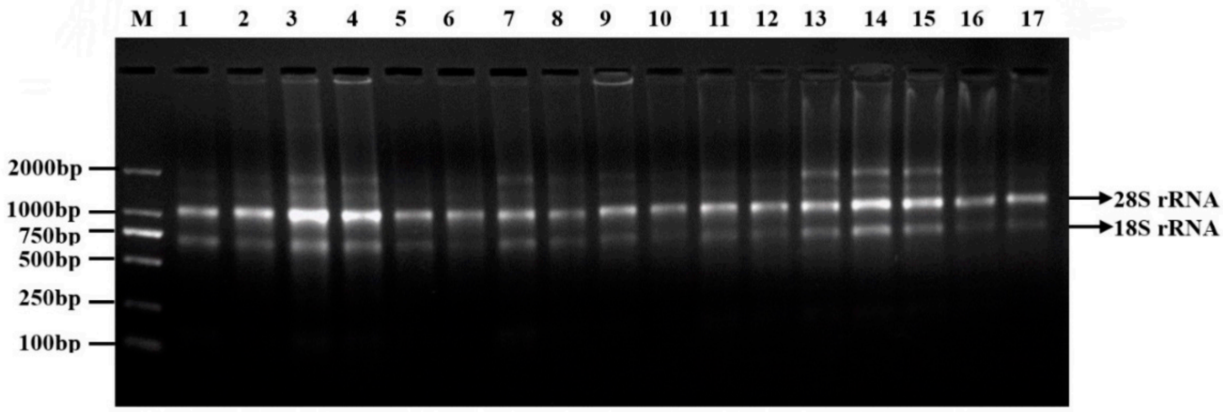


**Figure S1.** 1.2% agarose gel electrophoresis detection of total RNA extracted from lingonberry.

Note: M is 2000bp DNA Marker; 1-17 are random selection of total RNA from tested lingonberry samples.

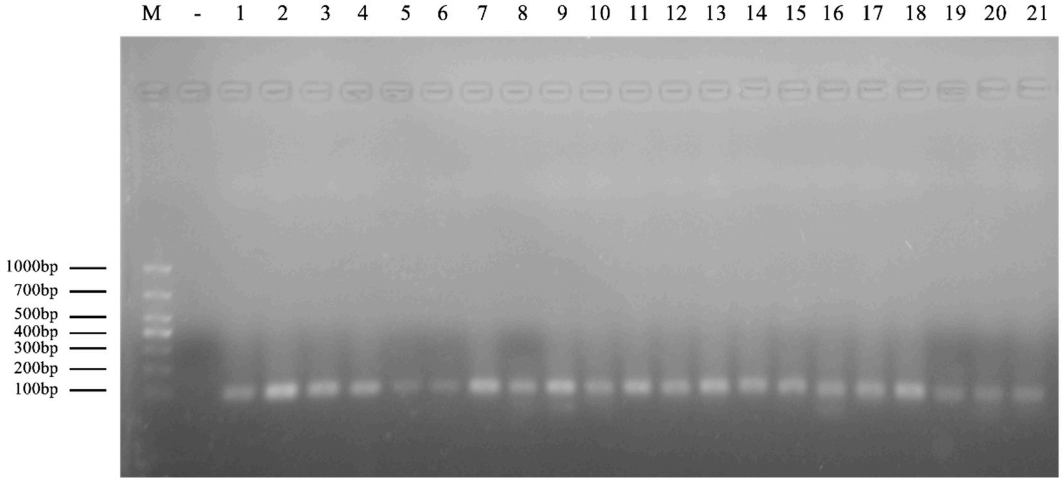


**Figure S2.** Products of qRT-PCR of 21 candidate reference genes.

Note: M: 1000bp DNA Marker; -: Negative control,

1-21: PCR Products (*Actin CL1167.Contig3_All, Actin CL2126 Contig2_All, Actin CL2172 Contig2_All, Actin CL2172.Contig3_All, Actin CL3559.Contig7_All, Actin CL494.Contig13_All, Actin CL5740.Contig1_All, Actin CL5740.Contig2_All, Actin CL5740.Contig5_All, Actin CL7856.Contig2_All, Actin Unigene12465_All, Actin Unigene20323_All, Actin Unigene23839_All, Actin Unigene6171_All, Chy Unigene26262_All, 18S rRNA CL5051.Contig1_All, Tub CL1466.Contig3_All, Tub CL1466.Contig7_All, Tub CL3192.Contig5_All, Tub CL7489.Contig2_All, Tub Unigene3128_All.*)
