# Supplementary material for: Transcriptome-Based Identification of the Optimal Reference Genes for Quantitative Real-Time Polymerase Chain Reaction Analyses of Lingonberry Fruits throughout the Growth Cycle
Source: Plants (Basel). 2023 Dec 16;12(24):4180. doi: 10.3390/plants12244180 (PMC10748091; doi:10.3390/plants12244180)
Supplement: Supplementary file 1 [file plants-12-04180-s001.zip › Table S1-plants.pdf]

Table S1. Non-significantly differentially expressed genes in lingonberries among 10 candidate gene families.

Gene	Transcript ID	R_FPKM	W_FPKM	G_FPKM	log_2(R_FPKM/ G_FPKM)	log_2(W_FPKM/ G_FPKM)	log_2(R_FPKM/ W_FPKM)
Actin	*CL1167.Contig3_All*	0.8334	0.4411	0.4053	1.0400	0.1221	0.9179
	CL1395.Contig5_All	2.1298	2.297	5.5324	-1.3772	-1.2682	-0.1090
	CL2126.Contig2_All	0.7996	2.0128	2.7986	-1.8074	-0.4755	-1.3319
	CL2172.Contig2_All	2.3576	0.6851	0.8533	1.4662	-0.3167	1.7829
	CL2172.Contig3_All	2.7502	1.1539	0.9539	1.5276	0.2746	1.2530
	CL3390.Contig4_All	1.7819	1.636	4.8493	-1.4444	-1.5676	0.1232
	CL3559.Contig7_All	2.0047	2.0123	4.6532	-1.2148	-1.2094	-0.0055
	CL4378.Contig1_All	54.1388	39.4773	18.8652	1.5209	1.0653	0.4556
	CL4850.Contig2_All	9.7241	5.7375	4.4036	1.1429	0.3817	0.7611
	CL494.Contig13_All	1.0239	1.6169	3.7289	-1.8647	-1.2055	-0.6592
	CL5229.Contig3_Al	5.646	7.1462	11.3399	-1.0061	-0.6662	-0.3400
	CL5740.Contig1_All	1.3244	0.6048	0.6291	1.0740	-0.0568	1.1308
	CL5740.Contig2_All	0.9473	0.2527	0.4204	1.1721	-0.7343	1.9064
	CL5740.Contig5_All	0.8091	0.2982	0.2511	1.6881	0.2480	1.4400
	CL6840.Contig1_All	45.6745	73.8838	113.8797	-1.3181	-0.6242	-0.6939
	CL7185.Contig1_All	1.4962	3.5282	4.5525	-1.6054	-0.3677	-1.2376
	CL8011.Contig1_All	4.2459	10.8325	15.6458	-1.8816	-0.5304	-1.3512
	Unigene10648_All	4.353	16.5439	10.9238	-1.3274	0.5988	-1.9262
	Unigene12465_All	0.675	1.3183	2.107	-1.6422	-0.6765	-0.9657
	Unigene15984_All	31.9788	21.2145	13.5045	1.2437	0.6516	0.5921
	Unigene17936_All	1.7729	2.0314	5.682	-1.6803	-1.4839	-0.1964
	Unigene18404_All	3.9142	10.4685	15.5823	-1.9931	-0.5739	-1.4193
	Unigene20323_All	1.032	2.2993	3.2932	-1.6741	-0.5183	-1.1558
	Unigene22378_All	32.1612	22.9379	15.6101	1.0428	0.5553	0.4876
	Unigene23839_All	0.6218	0.7745	1.7724	-1.5112	-1.1944	-0.3168
	Unigene24131_All	5.246	6.798	12.6972	-1.2752	-0.9013	-0.3739
	Unigene24295_All	5.6332	16.2485	16.4857	-1.5492	-0.0209	-1.5283

Table S1. *Cont.*

Gene	Transcript ID	R_FPKM	W_FPKM	G_FPKM	log_2(R_FPKM/G_FPKM)	log_2(W_FPKM/G_FPKM)	log_2(R_FPKM/W_FPKM)
Actin	*Unigene469_All*	3.6164	4.6717	7.7875	-1.1066	-0.7372	-0.3694
	Unigene4870_All	125.4805	80.1892	43.7176	1.5212	0.8752	0.6460
	Unigene6171_All	1.3973	2.4301	3.5309	-1.3374	-0.5390	-0.7984
	Unigene7378_All	48.4975	20.3828	13.2946	1.8671	0.6165	1.2506
Tub	*CL1466.Contig3_All*	4.9353	4.09	2.4546	1.0077	0.7366	0.2710
	CL1466.Contig7_All	1.979	4.7931	4.6206	-1.2233	0.0529	-1.2762
	CL1466.Contig9_All	7.5292	4.7383	3.5907	1.0682	0.4001	0.6681
	CL1656.Contig1_All	6.5721	2.2412	2.7214	1.2720	-0.2801	1.5521
	CL2137.Contig1_All	4.8444	2.5441	1.2602	1.9427	1.0135	0.9292
	CL3192.Contig4_All	4.8459	9.5367	16.3973	-1.7586	-0.7819	-0.9767
	CL3192.Contig5_All	0.419	0.8001	1.442	-1.7831	-0.8498	-0.9332
	CL3192.Contig9_All	33.0911	33.517	8.8942	1.8955	1.9140	-0.0185
	CL7163.Contig1_All	10.3902	7.2016	3.6798	1.4975	0.9687	0.5288
	CL7163.Contig2_All	6.7321	5.0146	3.3008	1.0282	0.6033	0.4249
	CL7489.Contig2_All	1.3349	1.7298	3.241	-1.2797	-0.9058	-0.3739
	Unigene15297_All	3.6548	7.0791	13.5818	-1.8938	-0.9400	-0.9538
	Unigene24354_All	22.1004	55.828	48.3005	-1.1280	0.2090	-1.3369
	Unigene3128_All	2.3748	2.1145	5.1695	-1.1222	-1.2897	0.1675
	Unigene3627_All	29.3621	31.4436	13.2787	1.1448	1.2437	-0.0988
	Unigene6908_All	6.5042	14.3043	15.8101	-1.2814	-0.1444	-1.1370
Tbp	*CL5036.Contig1_All*	1.9901	3.3199	4.9984	-1.3286	-0.5903	-0.7383
Cyp	*CL1792.Contig2_All*	5.7131	8.4218	11.8632	-1.0542	-0.4943	-0.5599
	CL4189.Contig2_All	70.5761	168.8836	204.6606	-1.5360	-0.2772	-1.2588
	CL6149.Contig1_All	28.0076	63.0056	57.8194	-1.0457	0.1239	-1.1697
	Unigene24946_All	37.1774	30.5428	17.6026	1.0786	0.7950	0.2836
	Unigene7522_All	30.5437	32.7739	10.8645	1.4913	1.5929	-0.1017
Chy	*Unigene26262_All*	0.9312	0.2635	0.4187	1.1532	-0.6681	1.8213
GAPDH	*CL3106.Contig2_All*	14.5159	10.8998	4.9859	1.5417	1.1284	0.4133
	CL403.Contig3_All	85.651	56.2458	27.1197	1.6591	1.0524	0.6067
	CL403.Contig7_All	25.9444	15.7348	11.4078	1.1854	0.4639	0.7215
	CL403.Contig8_All	11.9848	5.2991	5.633	1.0892	-0.0882	1.1774
	Unigene11802_All	10.9751	4.6015	5.0877	1.1091	-0.1449	1.2541

Table S1. *Cont.*

Gene	Transcript ID	R_FPKM	W_FPKM	G_FPKM	log_2(R_FPKM/ G_FPKM)	log_2(W_FPKM/ G_FPKM)	log_2(R_FPKM/ W_FPKM)
GAPDH	*Unigene11804_All*	29.004	15.3624	13.8181	1.0697	0.1528	0.9168
18S rRNA	*CL5051.Contig1_All*	1.5278	3.1211	4.2146	-1.4639	-0.4333	-1.0306
EF-1α	*CL1344.Contig3_All*	113.2963	89.4631	52.3921	1.1127	0.7719	0.3407
	CL1344.Contig5_All	12.3474	8.6156	4.9415	1.3212	0.8020	0.5192
EIF	*CL3626.Contig1_All*	4.6906	3.7459	1.5973	1.5541	1.2297	0.3245
	CL3626.Contig2_All	8.0723	3.7621	2.1999	1.8755	0.7741	1.1014
	CL3766.Contig1_All	53.69	120.916	192.9366	-1.8454	-0.6741	-1.1713
	CL3766.Contig2_All	90.8429	110.4604	269.2429	-1.5675	-1.2854	-0.2821
	CL4277.Contig2_All	7.2336	3.9381	2.8391	1.3493	0.4721	0.8772
	CL6019.Contig2_All	37.5478	25.2233	17.2381	1.1231	0.5492	0.5740
	CL6019.Contig3_All	11.2844	7.7127	5.068	1.1548	0.6058	0.5490
	CL6910.Contig2_All	13.6323	27.96	35.3978	-1.3766	-0.3403	-1.0363
	Unigene1121_All	9.53	14.3619	4.1816	1.1884	1.7801	-0.5917
	Unigene25060_All	5.5034	12.3342	14.4752	-1.3952	-0.2309	-1.1643
	Unigene4669_All	7.0338	10.0275	14.5361	-1.0473	-0.5357	-0.5116
