# Supplementary material for: Transcriptome-Based Identification of the Optimal Reference Genes for Quantitative Real-Time Polymerase Chain Reaction Analyses of Lingonberry Fruits throughout the Growth Cycle
Source: Plants (Basel). 2023 Dec 16;12(24):4180. doi: 10.3390/plants12244180 (PMC10748091; doi:10.3390/plants12244180)
Supplement: Supplementary file 1 [file plants-12-04180-s001.zip › Table S2-plants.pdf]

Table S2. Average Ct value, standard deviation (SD) and coefficient of variation (CV) of candidate internal reference genes in all lingonberry samples.

Gene	Mean Ct	Standard deviation	Coefficient of variation
18S rRNA CL5051.Contig1	29.98	1.37	4.48
Actin CL1167.Contig3	28.68	1.44	5.04
Actin CL2126.Contig2	29.04	1.41	4.90
Actin CL2172.Contig2	23.29	0.97	4.22
Actin CL2172.Contig3	25.19	1.40	5.46
Actin CL3559.Contig7	30.64	1.10	3.66
Actin CL494.Contig13	27.67	1.26	4.59
Actin CL5740.Contig1	28.49	1.49	5.32
Actin CL5740.Contig2	30.34	1.37	4.58
Actin CL5740.Contig5	28.46	1.62	5.75
Actin CL7856.Contig2	32.03	1.33	4.26
Actin Unigene12465	26.96	0.89	3.28
Actin Unigene20323	31.73	1.08	3.43
Actin Unigene23839	30.79	1.17	3.87
Actin Unigene6171	31.54	1.22	3.96
Chy Unigene26262	31.76	1.42	4.56
Tub CL1466.Contig3	29.95	1.42	4.77
Tub CL1466.Contig7	32.29	1.06	3.31
Tub CL3192.Contig5	29.57	1.68	5.76
Tub CL7489.Contig2	29.55	2.12	7.36
Tub Unigene3128	29.77	1.28	4.32
