# Supplementary material for: Transcriptome-Based Identification of the Optimal Reference Genes for Quantitative Real-Time Polymerase Chain Reaction Analyses of Lingonberry Fruits throughout the Growth Cycle
Source: Plants (Basel). 2023 Dec 16;12(24):4180. doi: 10.3390/plants12244180 (PMC10748091; doi:10.3390/plants12244180)

Table S3. The melting curves of 21 candidate reference mRNA genes.

Symbol	Dissociation curve
Actin CL1167.Contig3_All	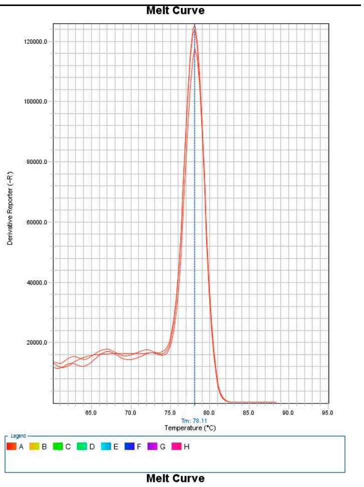
Actin CL2126.Contig2_All	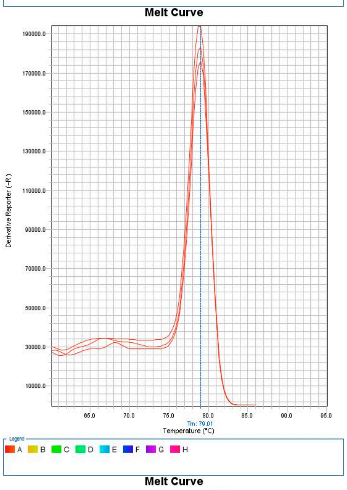
Actin CL2172.Contig2_All	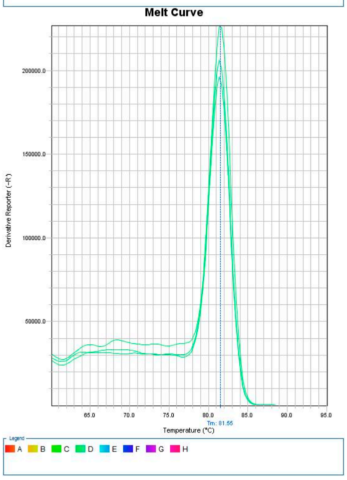

Actin CL2172.Contig3_All

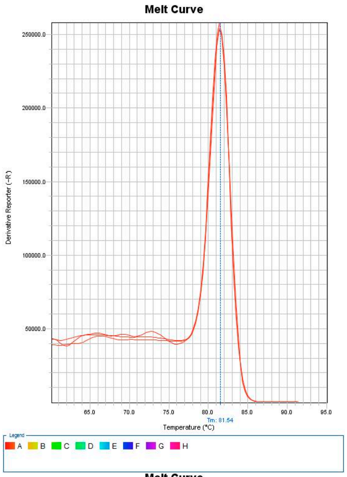

Actin CL3559.Contig7_All

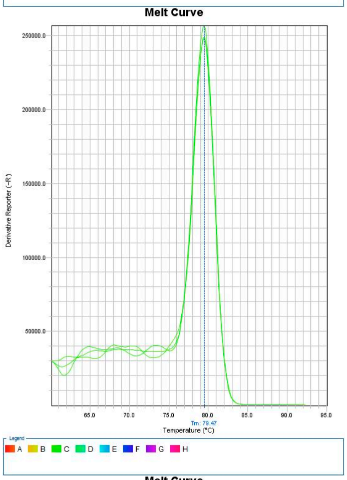

Actin CL494.Contig13_All

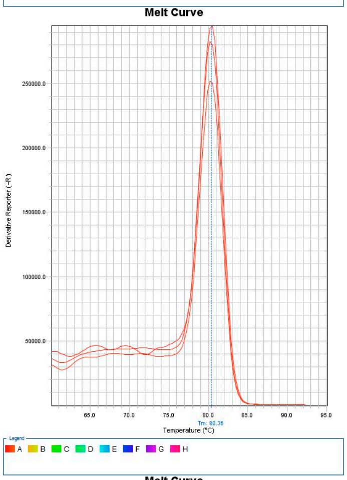

Actin CL5740.Contig1_All

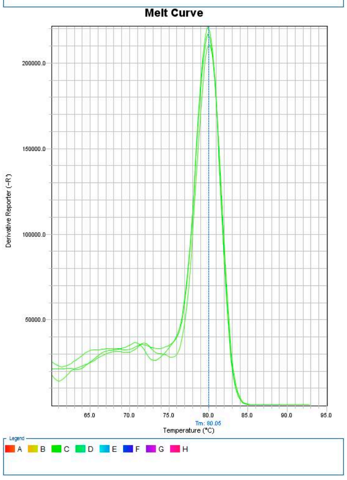

Actin CL5740.Contig2_All

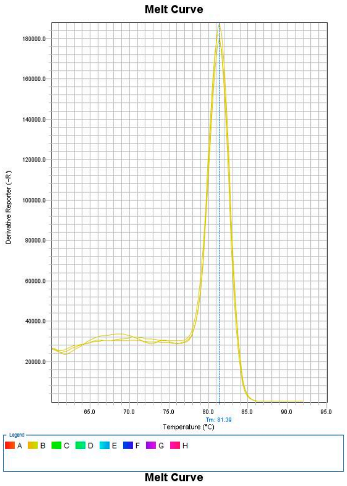

Actin CL5740.Contig5_All

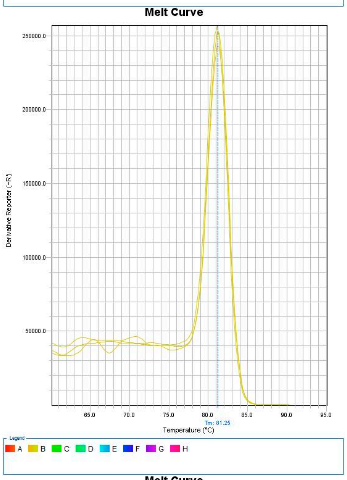

Actin CL7856.Contig2_All

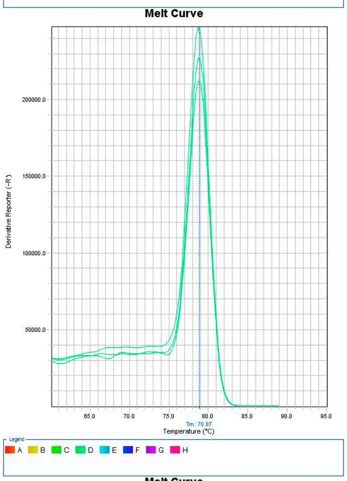

Actin Unigene12465_All

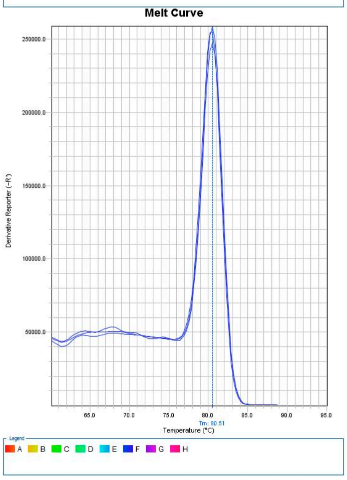

Actin Unigene20323_All

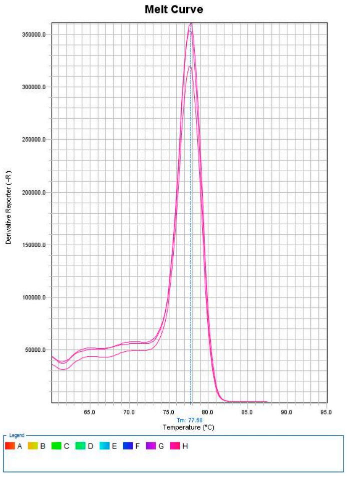

Actin Unigene23839_All

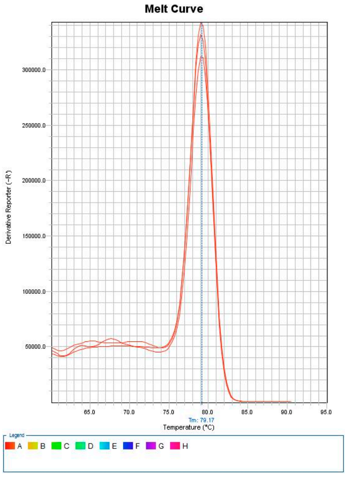

Actin Unigene6171_All

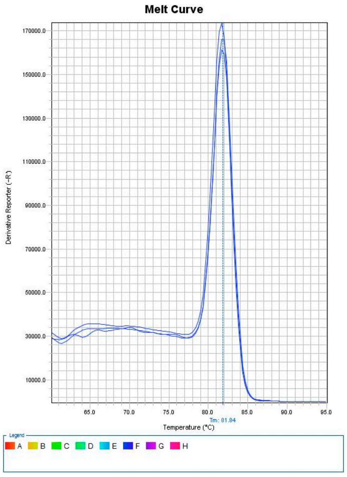

Chy Unigene26262_All

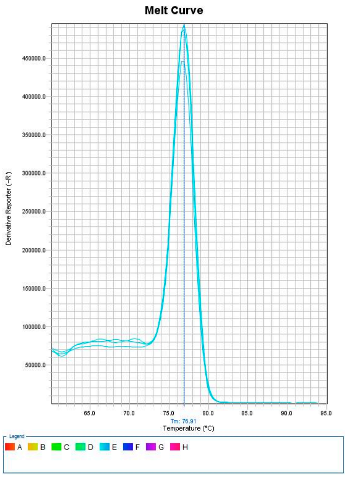

18S rRNA CL5051.Contig1_All

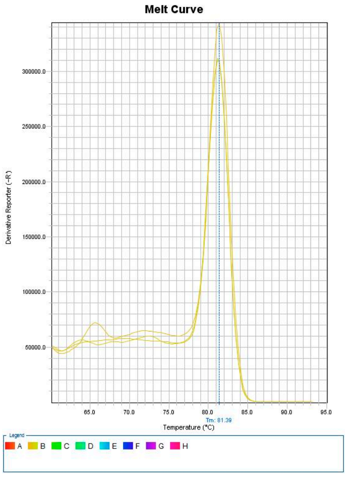

Tub CL1466.Contig3_All

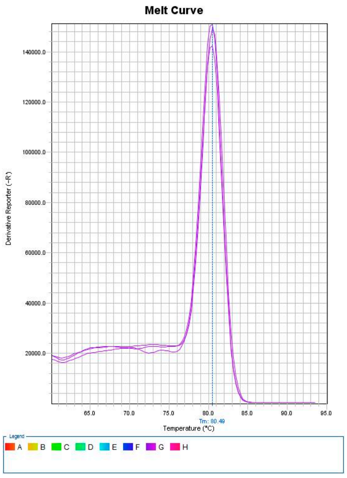

Tub CL1466.Contig7_All

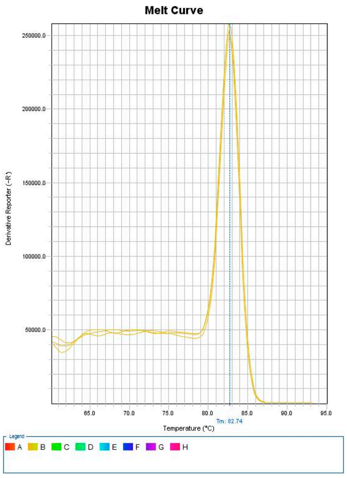

Tub CL3192.Contig5_All

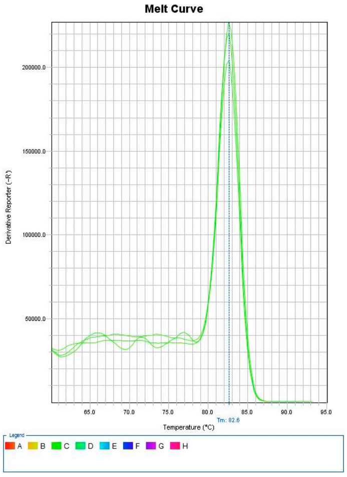

Tub CL7489.Contig2_All

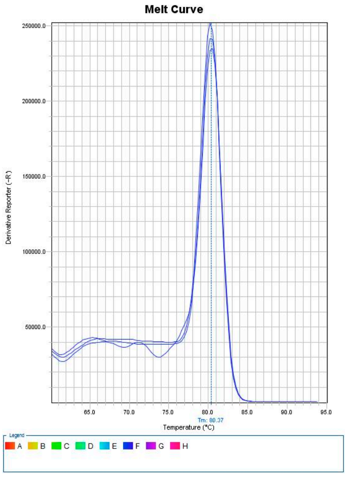

Tub Unigene3128_All

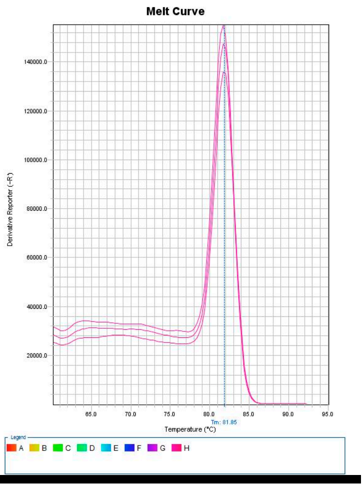

Supplement: Supplementary file 1 [file plants-12-04180-s001.zip › Table S3-plants.pdf]
